# The discrepancy between objective and subjective assessments of catastrophic health expenditure: evidence from China

**DOI:** 10.1093/heapol/czae115

**Published:** 2024-12-02

**Authors:** Bingqing Guo, Chaojie Liu, Qiang Yao

**Affiliations:** School of Public Health, Li Ka Shing Faculty of Medicine, The University of Hong Kong, No.7 Sasson Road, Pok Fu Lam, Hong Kong SAR, 999077, China; School of Psychology and Public Health, La Trobe University, 1 Kingsbury Dr, Melbourne, VIC 3086, Australia; School of Political Science and Public Administration, Wuhan University, No.299, Bayi Road, Wuchang District, Wuhan, Hubei, 430072, China

**Keywords:** sustainable development goal, universal health care, catastrophic health expenditure, China

## Abstract

The pro-rich nature of catastrophic health expenditure (CHE) indicators has garnered criticism, inspiring the exploration of the subjective approach as a complementary method. However, no studies have examined the discrepancy between subjective and objective approaches. Employing data from the Chinese Social Survey (CSS) 2013–2021 waves, we analysed the discrepancy between objective and subjective CHE and its associated socioeconomic factors using logit regression modelling. Overall, self-rating generated higher CHE incidence (28.35% to 33.72%) compared to objective indicators (9.92% to 21.97%). Objective indicators did not support 17.57% to 23.90% of self-rated cases of household CHE, while 2.73% to 8.42% of households classified with CHE by objective indicators did not self-rate with CHE. The normative subsistence spending indicator showed the least consistency with self-rating (70.66% to 74.28%), while the budget share method produced the most consistent estimation (72.73% to 76.10%). Living with elderly and young children [adjusted odds ratios (AOR): 1.069 to 1.169, *P* < 0.1], lower educational attainment (AOR: 1.106 to 1.225, *P* < 0.1), lower income (AOR: 1.394 to 2.062, *P* < 0.01), and lower perceived social class (AOR: 1.537 to 2.801, *P* < 0.05) were associated with higher odds of self-rated CHE without support from objective indicators. Conversely, low socioeconomic status (AOR: 0.324 to 0.819, *P* < 0.1) was associated with lower odds of missing CHE cases classified by objective indicators in self-rating. The commonly used objective indicators for assessing CHE may attract doubts about their fairness from socioeconomically disadvantaged people. The CHE subjective approach can be adopted as a complementary measure to monitor financial risk protection.

Key messages

- The self-rating method produced a higher incidence of catastrophic health expenditure (CHE) than traditional objective CHE indicators.
- The normative subsistence spending method is least consistent with the self-rating method, but the budget share method is most consistent.
- The inconsistencies between self-rated and objective CHE results reflect the potential inequities in CHE monitoring: self-rated CHE without support by objective indicators is associated with low socioeconomic status. However, CHE without backup from self-rating is associated with high socioeconomic status.

## Introduction

Financial risk protection, meaning that all people receive essential health services without being exposed to financial hardship, is one of the critical goals of universal health coverage and the sustainable development goals (World Health Organization 2013, World Health Organization, World Bank 2015). Many countries are trying to reduce household financial hardship resulting from exorbitant healthcare expenditures through financial protection systems. Accurately estimating the financial hardship due to healthcare expenditures is vital for evaluating the performance of such systems Many indicators have been developed to measure financial hardship, including the official sustainable development goals indicator—catastrophic health expenditure (CHE), which measures out-of-pocket (OOP) healthcare expenditure that reduces the ability of a household to spend on other essential items (Wagstaff and van Doorslaer 2003, Xu 2005). Integrated indicators were also developed, including percentages of households incurring immiserating, impoverishing, catastrophic, and zero OOP payments (Wagstaff and Eozenou 2014). Medical debt and income loss have also been used in some country-specific studies (Finkelstein et al. 2012). CHE is the most popular among current indicators due to its low data requirements, ease of calculation and interpretation, and high acceptance by stakeholders (Grépin et al. 2020).

The past four decades have seen the evolution of CHE measurements. Initially, CHE was defined as a high absolute amount of OOP healthcare expenditure, e.g. $5000 per year, as established by Birnbaum’s study (Fairfieldsonn 1980). A significant weakness of this approach is that the specified amount represents a heavy burden for poor households but not for wealthier ones. Consequently, some studies have defined CHE in terms of healthcare expenditures that comprise a high percentage of total available resources or capacity to pay. These relative-amount approaches have become dominant and are endorsed by many influential international organisations, such as the World Health Organization (WHO), the United Nations, and the World Bank (WB) (Xu 2005, Wagstaff and Eozenou 2014, World Health Organization, World Bank 2015).

However, there is no consensus on the threshold or the denominator. Two common approaches for setting the denominator are total household budget share and capacity to pay. The budget share approach calculates OOP healthcare expenditure as a percentage of household income or expenditure (Cylus et al. 2018). This method is easy to understand and applicable; the required data are readily available in most countries. As a result, it has been popular in studies focusing on low- and middle-income countries (LMICs), including India, Afghanistan, and Nigeria (Dastan et al. 2021, Dwivedi et al. 2021, Edeh 2022, Gaddam and Rao 2023, Opeloyeru and Lawanson 2023). Common thresholds are typically set at 2.5%, 10%, 25%, or 40% (Wagstaff and van Doorslaer 2003, Cylus et al. 2018). When income is used as a denominator, it assumes that people can allocate all available resources to health, which is often unrealistic. Although considered a better denominator due to its availability in LMICs, the expenditure-based method is also flawed. For example, individuals may cope with healthcare bills by incurring debt or receiving assistance (Flores et al. 2008, O’Donnell et al. 2008).

The capacity-to-pay approach addresses these limitations by replacing the denominator with household spending net of essential expenditures. A large body of literature defines essential spending as actual food expenditure (Zhong et al. 2020). To account for potential under- or over-spending, a method for estimating partial normative food spending has been proposed, reflecting households’ food expenditures in the middle socioeconomic range (Xu 2005). This method calculates the average food expenditure of households whose food spending share falls within the 45th to 55th percentile range, termed ‘partial normative food spending’. Capacity to pay is measured as total expenditure minus partial normative food spending if a household’s total spending exceeds this level; otherwise, it is calculated as total spending minus actual food spending (Xu 2005). The actual and partial normative food spending methods have been widely utilised across all income groups, including LMICs (e.g. India, Vietnam) and high-income countries (HICs) (e.g. South Korea and Canada). Some researchers argue that essential needs should encompass food, housing, and utilities, thus proposing the normative subsistence spending method (Thomson et al. 2018). This method is primarily used in European HICs, including Austria, Czechia, and France (Cylus et al. 2018).

Despite extensive efforts to improve the CHE measurements, current CHE indicators have significant limitations. Previous studies suggest that common objective CHE indicators underestimate the financial hardship experienced by poor households, whose essential and healthcare needs are constrained. This has been criticised as a ‘pro-rich’ bias. For example, studies in Burkina Faso, China, India, Indonesia, Malaysia, Nepal, and the Philippines found that the wealthiest households had a higher rate of CHE compared to the poorest households, as estimated by the budget share and the actual food method (Van Doorslaer et al. 2007, Trakinsky et al. 2020). These objective indicators also apply a universal threshold, ignoring the fact that households’ capacity to pay depends on various individual circumstances, such as coping mechanisms and anticipated future income (Wagstaff 2019).

These limitations underscore the need for complementary approaches and highlight the importance of including subjective measures. Subjective measures are considered valuable complements to objective measures in the social sciences because they reflect individual differences (Diener et al. 1999, World Health Organization 2024). Researchers have begun to introduce subjective measures to assess financial hardship due to OOP healthcare expenditures, although these measures are not fully aligned with the concept of CHE (Markman and Luce 2010, Valtorta and Hanratty 2013, Liu et al. 2021). Most of these studies have focused on households with critically ill patients, such as those with cancer, which may not represent the general population (Markman and Luce 2010, Valtorta and Hanratty 2013). Therefore, exploration of subjective approaches to CHE estimation remains limited. Further studies are needed to determine the complementary value of subjective methods alongside objective ones.

To address the research gap, the current study aims to examine how Chinese people perceive the financial burden of healthcare and identify any discrepancies that may exist between objective and subjective assessments of CHE. In this study, the term ‘objective CHE approach’ refers to methods in which CHE is computed using documented or recalled household income, expenditures, and OOP healthcare expenditures. Conversely, the term ‘subjective approach’ involves measuring CHE by relying on self-reported instances of unaffordable high OOP healthcare expenditures. Additionally, this study explores factors contributing to these discrepancies in CHE estimations. To the best of our knowledge, this is the first study to empirically analyse the discrepancies between objective and subjective assessments of CHE. This research highlights the importance of a more comprehensive approach to measuring CHE.

## Methods

### Data

Data were extracted from the 2013, 2017, 2019, and 2021 waves of the Chinese Social Survey (CSS). Four waves of the CSS (2006, 2008, 2011, and 2015) were excluded due to the limited availability of the required data on OOP healthcare expenditure or self-rated CHE in the past year. The CSS is a series of repeated cross-sectional surveys initiated in 2006 and coordinated by the Institute of Sociology at the Chinese Academy of Social Sciences. It drew samples from 604 rural villages and urban communities in 151 districts/counties across 31 provinces in mainland China using the sampling frame of the fifth and sixth population censuses (Chinese Academy of Social Sciences 2023). The trained investigators, who worked in teams, collected the data. The participating households from sample villages/communities were identified first using a mobile spatial application. The investigators then randomly selected one adult member from each household to answer questions face-to-face. The interviewee’s responses were recorded using the Computer Aided Surveying System–Computer Assisted Personal Interview System, an application designed by the CSS working group. A quality assurance process was conducted through random manual checking and re-interviewing. All participants were informed about the purpose and protocol of the survey. Written informed consent was obtained from each respondent. The current study was reviewed and approved by the Biomedical Ethics Committee of the corresponding author’s institution.

The CSS initially included 40 768 households: 10 206 in 2013, 10 143 in 2017, 10 283 in 2019, and 10 136 in 2021. We excluded 30 households (0.07%) whose respondents were <18 or >69 years old, as they fell outside the eligible age range of respondents according to the survey manual of the CSS (Chinese Academy of Social Sciences 2023). Our analyses were all based on households that sought care and incurred OOP healthcare expenditures in the past year. To ensure comparability between the self-rated and objective CHE indicators, we excluded 970 households (2.38%) that self-rated their healthcare expenditure as unaffordable but reported no OOP healthcare expenditures in the past year (supplementary Figs S1 and S2, see online supplementary material). Finally, we included 39 768 households in our analysis, comprising 10 014 from 2013, 9912 from 2017, 10 016 from 2019, and 9826 from 2021. Supplementary Fig. S1 further outlines the process used to determine the sample size for each data analysis.

### Measurements

#### Dependent variables

Discrepancies between objective and subjective CHE indicators were a major focus of this study, serving as the dependent variables. To construct these dependent variables, we first developed objective and subjective indicators to measure CHE.

Four objective CHE indicators, which are widely used globally, were constructed (Table 1). (i) Budget share indicator: the share of OOP healthcare expenditure in total household expenditure (threshold >25%) (Wagstaff and van Doorslaer 2003), (ii) Actual food spending indicator: OOP healthcare expenditure as a proportion of actual non-food monetary spending (threshold >40%) (World Health Organization, World Bank 2015). (iii) Partial normative food spending indicator: OOP healthcare expenditure as a proportion of non-food expenditure estimated through partial normative food spending (threshold >40%; calculation details in Table 1) (Xu 2005, Cylus et al. 2018). (iv) Normative subsistence spending indicator: OOP healthcare expenditure as a proportion of non-normative subsistence (food, housing, and utilities; threshold >40%; calculation details in Table 1) (Cylus et al. 2018, Thomson et al. 2018). These four indicators are among the most widely recognised measures of CHE globally, endorsed by organisations such as the WHO and the WB (Xu et al. 2003, Wagstaff and Eozenou 2014, Cylus et al. 2018).

**Table 1.** Indicators measuring household CHE

Approach	Method	Numerator	Denominator	CHE threshold
Budget share	Budget share	OOP healthcare expenditure	Total household expenditure	>25%
Capacity to pay	Actual food spending	OOP healthcare expenditure	Total household expenditure minus actual food spending	>40%
	Partial normative food spending	OOP healthcare expenditure	Total household expenditure minus standard food spending that corresponds to the average food expenditure of households whose food share is in the 45th to 55th percentile range, adjusted for (household size) ^0.56^; or total household expenditure minus actual food spending for those whose total expenditure is less than the standard food spending.	>40%
	Normative subsistence spending	OOP healthcare expenditure	Total household expenditure minus standard subsistence spending corresponds to the average subsistence spending among households whose total expenditures are in the 25th and 35th percentile range, adjusted for (household size) ^0.56^. For very poor households whose capacity-to-pay is negative, they are identified as having CHE if their incurr any OOP.	>40%

Each CHE indicator has its own strengths and limitations. For instance, the budget share method is often criticised for underestimating the CHE of poor households, but it remains the simplest to implement (Grépin et al. 2020). The normative subsistence spending indicator broadens the definition of essential needs beyond just food spending, although its use is constrained by data availability (Cylus et al. 2018). By incorporating multiple indicators in this study, we aimed to gain a comprehensive understanding of the discrepancies between commonly used objective indicators and self-rated indicators. Additionally, utilising multiple indicators allowed us to assess whether the observed discrepancies are consistent across different indicators or specific to a single one.

The CSS questionnaire included a section on the household’s economic situation, where respondents were asked to list their household expenditures for various items over the past year, including food, clothing, rental and mortgage payments, furniture and appliances, travel and transport, automobiles, utilities (gas, water, electricity), telecommunications, education, OOP expenditure for healthcare, cultural and recreational activities, care for family members and relatives, down payments for housing, and other expenditures (the full list of questions, time frames, and sequences are listed in supplementary Table S1, see online supplementary material). We then calculated the subsistence and total expenditures by summing the relevant items (Xie et al. 2017).

A binary variable, self-rated CHE, was constructed to measure CHE using a subjective approach. In the section of the CSS surveys regarding respondents’ self-evaluation of their life situation, respondents were asked to rate whether their OOP healthcare expenditure over the past year was too high (yes or no) to be affordable (the full list of questions, time frames, and sequences are listed in supplementary Table S1).

We then constructed eight binary variables as the dependent variables for the regression models. As mentioned earlier, we developed four objective CHE indicators and measured the discrepancies between each objective CHE indicator and the self-rated CHE indicator. Two types of discrepancies were calculated in terms of their number and percentages. The first type refers to instances of self-rated CHE that were not supported by the objective indicators. The second type pertains to incidents of household CHE classified by an objective indicator but not backed by self-rating. The former identifies those who felt the burden of CHE but whose experiences were not verified by the objective CHE indicators, while the latter identifies individuals who did not feel the burden of CHE despite being classified as experiencing CHE by the objective indicators.

For the specific dependent variables used in each regression model see supplementary Table S2 in the online supplementary material.

#### Independent variables

The independent variables are shown in Table 2. We selected specific independent variables based on existing systematic reviews on CHE and categorised them into four categories, including demographic, needs, socioeconomic, and regional factors (Doshmangir et al. 2021, Borde et al. 2022, Eze et al. 2022). The demographic factor included household size and gender ratio (Doshmangir et al. 2021). Household size was measured by the reported number of family members who lived together or were financially dependent on each other at the time period of the survey. Living with a household member >65 or <5 years old was considered a proxy for healthcare needs, as the elderly and young children typically require more healthcare (Borde et al. 2022, Eze et al. 2022, Mo et al. 2024). The socioeconomic factor contained several variables: highest educational attainment, per capita household income, perceived social class, the proportion of non-working age members, unemployment, residency (urban/rural), household registration, and health insurance (Doshmangir et al. 2021, Borde et al. 2022). The regional factor included the location and the per capita governmental budget on health of the province where the respondents resided (Eze et al. 2022, Jia et al. 2023, Michael et al. 2023). We also included year dummy variables. Household size, gender ratio, per capita household income, proportion of non-working-age members, and per capita governmental budget on health were transformed into categorical variables, with cutoff points defined by the x-tile syntax in each wave of the CSS (StataCorp 2021). The cutoff points are presented in supplementary Table S3, see online supplementary material. This transformation helps mitigate limitations associated with assuming a linear relationship between dependent and independent variables (James et al. 2021).

**Table 2.** Household characteristics

Variables	Measures	2013 (*n* = 10 014)	2017 (*n*= 9 912)	2019 (*n*= 10 016)	2021 (*n*= 9 826)	Total (*n* = 39 768)
		*n* (%)	*n* (%)	*n* (%)	*n* (%)	*n* (%)
Demographic factor						
Household size (chi-squared = 2700.00; *P* < 0.01)^a^						
Number of people living in a household	Small (ref.)	3 594 (35.89)	3 393 (34.23)	5 189 (51.81)	5 406 (55.02)	17 582 (44.21)
	Middle	4 117 (41.11)	4 078 (41.14)	1 814 (18.11)	1 723 (17.54)	11 732 (29.50)
	Large	2 303 (23.00)	2 441 (24.63)	3 013 (30.08)	2 697 (27.45)	10 454 (26.29)
Gender ratio (chi-squared = 30.33; *P* < 0.01)						
Proportion of female members in a household	Lower (ref.)	3 581 (35.76)	3 482 (35.13)	3 468 (34.62)	3 303 (33.61)	13 834 (34.79)
	Middle	3 357 (33.52)	3 405 (34.35)	3 230 (32.25)	3 322 (33.81)	13 314 (33.48)
	Higher	3 076 (30.72)	3 025 (30.52)	3 318 (33.13)	3 201 (32.58)	12 620 (31.73)
Needs factor						
Aged care need (chi-squared = 108.05; *P* < 0.01)						
Household with at least a member aged ≥65 years	No (ref.)	7 235 (72.25)	6 862 (69.23)	6 712 (67.01)	6 478 (65.93)	27 287 (68.62)
	Yes	2 779 (27.75)	3 050 (30.77)	3 304 (32.99)	3 348 (34.07)	12 481 (31.38)
Childcare need (chi-squared = 10.65; *P* < 0.05)						
Household with at least a member aged ≤5 years	No (ref.)	7 417 (74.07)	7 252 (73.16)	7 318 (73.06)	7 353 (74.83)	29 340 (73.78)
	Yes	2 597 (25.93)	2 660 (26.84)	2 698 (26.94)	2 473 (25.17)	10 428 (26.22)
Socioeconomic factor						
Highest educational attainment (chi-squared = 792.20; *P* < 0.01)						
Highest educational qualification attained by all household members	Up to primary school	757 (7.56)	867 (8.93)	506 (5.05)	429 (4.37)	2 559 (6.47)
	Middle school	4 881 (48.74)	4 706 (48.49)	4 127 (41.20)	3 687 (37.53)	17 401 (43.99)
	Vocational training	2 381 (23.78)	2 221 (22.88)	2 687 (26.83)	2 856 (29.07)	10 145 (25.64)
	Tertiary degree (Ref.)	1 995 (19.92)	1 912 (19.70)	2 696 (26.92)	2 853 (29.04)	9 456 (23.90)
Per capita household income (chi-squared = 22.93; *P* < 0.05)						
Annual household income per capita	Lowest	1 855 (19.91)	1 891 (19.97)	1 882 (19.91)	1 882 (20.05)	7 510 (19.96)
	Low	1 860 (19.96)	1 956 (20.65)	1 881 (19.90)	1 989 (21.19)	7 686 (20.43)
	Middle	1 968 (21.12)	1 826 (19.28)	1 897 (20.07)	1 763 (18.78)	7 454 (19.81)
	High	1 836 (19.70)	1 901 (20.07)	1 887 (19.96)	1 855 (19.76)	7 479 (19.88)
	Highest (ref.)	1 799 (19.31)	1 897 (20.03)	1 907 (20.17)	1 897 (20.21)	7 500 (19.93)
Perceived social class (chi-squared = 667.41; *P* < 0.01)						
Self-rating on social class	Upper (Ref.)	48 (0.49)	27 (0.27)	85 (0.86)	76 (0.78)	236 (0.60)
	Middle	7 646 (77.64)	6 370 (64.63)	7 562 (76.56)	7 448 (76.85)	29 026 (73.91)
	Lower	2 154 (21.87)	3 459 (35.10)	2 230 (22.58)	2 168 (22.37)	10 011 (25.49)
Proportion of non-working age members (chi-squared = 272.86; *P* < 0.01)						
Proportion of household members aged ≥65 years and ≤14 years	Lower (Ref.)	3 457 (34.52)	3 311 (33.40)	3 411 (34.06)	3 376 (34.36)	13 555 (34.09)
	Middle	4 083 (40.77)	3 823 (38.57)	3 349 (33.44)	3 240 (32.97)	14 495 (36.45)
	Higher	2 474 (24.71)	2 778 (28.03)	3 256 (32.51)	3 210 (32.67)	11 718 (29.47)
Unemployment (chi-squared = 863.01; *P* < 0.01)						
Unemployment of household members in working age	No (ref.)	6 198 (63.68)	4 126 (45.32)	4 383 (49.60)	3 913 (45.28)	18 620 (51.27)
	Yes	3 535 (36.32)	4 978 (54.68)	4 454 (50.40)	4 729 (54.72)	17 696 (48.73)
Residency (chi-squared = 525.48; *P* < 0.01)						
Location of current household	Rural	5 325 (54.90)	5 763 (58.48)	4 361 (43.54)	5 490 (55.88)	18 454 (46.85)
	Urban (Ref.)	4 374 (45.10)	4 091 (41.52)	5 655 (56.46)	4 334 (44.12)	20 939 (53.15)
Household registration (chi-squared = 27.69; *P* < 0.01)						
Alignment (at the prefecture level) between current household location and household registration is known as *Hukou*	Aligned (ref.)	8 841 (88.45)	8 819 (89.08)	8 785 (87.83)	8 512 (86.75)	34 957 (88.03)
	Not-aligned	1 154 (11.55)	1 081 (10.92)	1 217 (12.17)	1 300 (13.25)	4 752 (11.97)
Health insurance^b^ (chi-squared = 3100.00; *P* < 0.01)						
Health insurance coverage of the respondent	None (ref.)	936 (9.35)	1 859 (18.76)	1 489 (14.87)	3 365 (34.25)	7 649 (19.23)
	UEBMI	1 654 (16.52)	1 578 (15.92)	1 825 (18.22)	1 374 (13.98)	6 431 (16.17)
	URBMI	956 (9.55)	975 (9.84)	1 001 (9.99)	795 (8.09)	3 727 (9.37)
	GMI	221 (2.21)	97 (0.98)	112 (1.12)	118 (1.20)	548 (1.38)
	NRCMS	6 042 (60.34)	5 036 (50.81)	4 783 (47.75)	3 371 (34.31)	19 232 (48.36)
	Others	205 (2.05)	367 (3.70)	806 (8.05)	803 (8.17)	2 181 (5.48)
Regional factor						
Per capita governmental budget on health (chi-squared = 139.90; *P* < 0.01)						
Per capita governmental budget on health in each province	Lower (ref.)	5 429 (54.21)	5 483 (55.88)	5 430 (54.21)	5 075 (51.65)	21 417 (53.99)
	Middle	3 508 (35.03)	3 052 (31.11)	3 100 (30.95)	3 512 (35.74)	13 172 (33.21)
	Higher	1 077 (10.75)	1 277 (13.01)	1 486 (14.84)	1 239 (12.61)	5 079 (12.80)
Provincial location^c^ (chi-squared = 3.83; *P* = 0.70)						
Geographical location of the province is classified by the China Health Statistical Yearbook.	Eastern (ref.)	4 173 (41.67)	3 981 (40.57)	4 176 (41.69)	4 059 (41.31)	16 389 (41.32)
	Central	3 152 (31.48)	3 138 (31.98)	3 144 (31.39)	3 136 (31.92)	12 570 (31.69)
	Western	2 689 (26.85)	2 693 (27.45)	2 696 (26.92)	2 631 (26.78)	10 709 (27.00)

a Notes: Pearson’s chi-square and *P*-value are reported in parentheses to present the distribution changes across years for each characteristic.

b UEBMI = Urban Employee Basic Medical Insurance; URBMI = Urban Resident Basic Medical Insurance; GMI = Government Medical Insurance; NRCMS = New Rural Cooperative Medical Scheme. This table presents the full sample.

c Eastern region includes Beijing, Tianjin, Hebei, Liaoning, Shanghai, Jiangsu, Zhejiang, Fujian, Shandong, Guangdong, and Hainan. Central region includes Shanxi, Jilin, Heilongjiang, Anhui, Jiangxi, Henan, Hubei, and Hunan. Western region includes Inner Mongolia, Chongqing, Guangxi, Sichuan, Guizhou, Yunnan, Tibet, Shaanxi, Gansu, Ningxia, and Xinjiang.

### Data analysis

Household characteristics were described and compared across the four waves of the CSS using chi-square tests. Multivariate logistic regression models were established to identify the factors associated with each dependent variable.

For the regression models, we initially considered multi-level modelling, as the independent variables included measurements at both the household and provincial levels. However, the intraclass correlation coefficients for the eight regression models ranged from 0.007 to 0.013 (supplementary Table S4, see online supplementary material), significantly below the commonly accepted threshold of 0.059 for justifying multi-level modelling (Cohen 1988). This indicated minimal clustering effects, supporting the use of single-level models. Furthermore, comparisons of Akaike information criterion (AIC) values showed no substantial differences between the single-level and multi-level models (supplementary Table S5, see online supplementary material). Therefore, we opted to use single-level models.

The analyses were performed using Stata 18.0 SE, and a two-sided *P*-value < 0.1 was considered statistically significant. Missing values were managed through pairwise deletion, with the proportion of excluded households due to missing values varying across models due to changes in the dependent variable, ranging from 11.38% (or 4 641) to 12.11% (or 4 938) (supplementary Fig. S1).

The funding sources play no role in research design, execution, data analysis, or the decision to submit the paper for publication.

## Results

### Characteristics of households

Table 2 shows the characteristics of the households. Overall, 17 582 (44.21%) households were classified as small-sized, 11 732 (29.50%) as medium-sized, and 10 454 (26.29%) as large-sized. A total of 12 481 households (31.38%) had elderly members, while 10 428 (26.22%) had young children. Only 9 456 households (23.90%) included a member with a tertiary degree. The vast majority, 29 026 households (73.91%), rated their social status as middle class. Additionally, 17 696 households (48.73%) reported unemployment among working-age members. Approximately half of the households, 18 454 (46.85%) households lived in rural areas, and 4752 households (11.97%) resided in a prefecture outside their household registration (Hukou). Furthermore, 7649 households (19.23%) were not enrolled in social health insurance. The participating households were biased toward the more densely populated eastern region, with 16 389 (41.32%) households, compared to the sparsely populated western region, which comprised 10 709 (27.00%) households.

From 2013 to 2021, notable shifts in household characteristics were observed (Table 2). The proportion of small households rose from 35.89% (3 594) to 55.02% (5 406). Households with elderly members increased from 27.75% (2 779) to 34.07% (3 348), while those with young children slightly decreased from 25.93% (2 597) to 25.17% (2473). Additionally, households with members holding tertiary degrees grew from 19.92% (1 995) to 29.04% (2 853), and those reporting unemployment among working-age members rose from 36.32% (3 535) to 54.72% (4 729). Households living outside their registered prefecture (Hukou) increased from 11.55% (1 154) to 13.25% (1 300), and those without social health insurance surged from 9.25% (936) to 34.25% (3 365). The geographical distribution of households across eastern, central, and western regions remained stable. These trends align with broader demographic changes in China, including declining fertility rates, rapid ageing, and increased enrolment in tertiary education (Ministry of Education of China 2022, Wang and Chen 2022, H-t et al. 2024).

### CHE incidence

**Figure 1.**
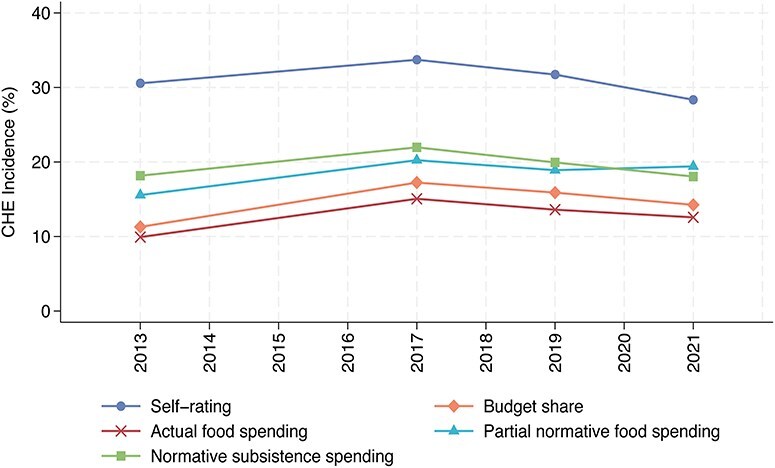
Catastrophic health expenditure incidence in China by year and estimation methods, 2013–2021.

The incidence of self-rated CHE increased from 30.56% in 2013 to 33.72% in 2017, before declining to 28.35% in 2021 (Fig. 1 and Table S6, see online supplementary material). Similar trends were observed across the four objective CHE indicators. For example, using the budget share method, the CHE incidence rose from 11.29% in 2013 to 17.24% in 2017, followed by a decline to 14.25% in 2021 (Fig. 1 and Table S6).

Overall, from 2013 to 2021, the CHE incidence captured by objective indicators was significantly lower than that reported through self-rating. Among the objective indicators, the actual food spending method yielded the lowest CHE incidence, ranging from 9.92% to 15.06%. In contrast, the normative subsistence spending method produced the highest CHE incidence, ranging from 18.05% to 21.97% (Fig. 1 and Table S6).

**Figure 2.**
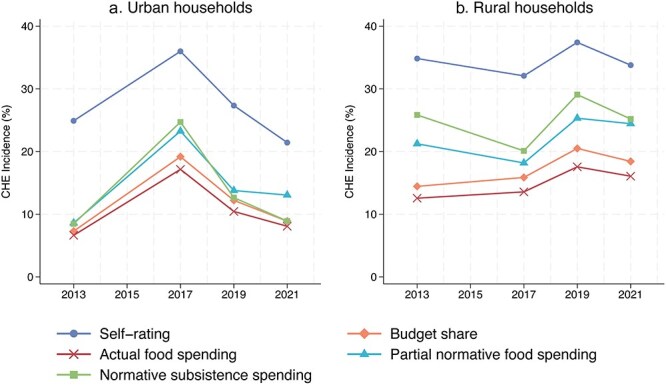
Catastrophic health expenditure incidence in China among (**a**) urban households and (**b**) rural households, by year and estimation methods, 2013–2021.

From 2013 to 2021, CHE incidence was generally higher among rural households compared to their urban counterparts, with the exception of 2017 (Fig. 2 and Table S6). For urban households, CHE incidence increased from 2013 to 2017, followed by a decline in subsequent waves. In contrast, rural households experienced a decrease in CHE incidence in 2017, followed by an increase in 2019, and then a subsequent drop in 2021 (Fig. 2 and Table S6).

### Discrepancies in CHE estimations

**Figure 3.**
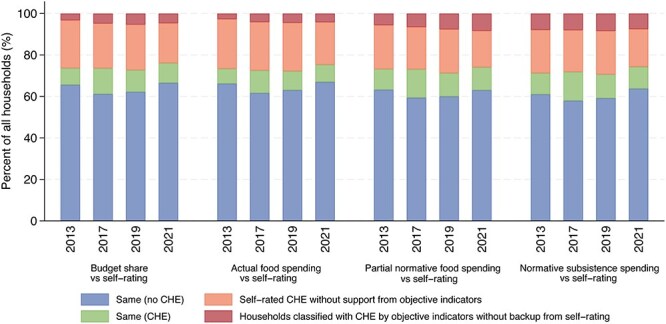
Consistency in estimations of household catastrophic health expenditure between self-rating and the objective indicators in China, 2013–2021.

Figure 3 shows that the objective indicators did not substantiate ∼20% (ranging from 17.57% to 23.90%) of self-rated CHE cases (for details see Table S7, see online supplementary material). The percentage of households classified with CHE by the objective indicators without backup from self-rating was relatively small, ranging from 2.73% to 8.42%. The normative subsistence spending method was slightly less likely to miss self-rated cases of CHE (18.21% to 20.92%) but slightly more likely to identify CHE cases without backup from self-rating (7.51% to 8.42%) compared to other indicators. By contrast, the actual food spending method was slightly more likely to miss self-rated CHE (20.44% to 23.90%) but slightly less likely to identify CHE cases without backup from self-rating (2.73% to 4.47%), compared with other indicators.

Figure 3 shows that ∼57.94% to 66.99% of households were deemed to have no CHE, compared with 7.22% to 13.91% of households having CHE, as identified by both self-rating and objective indicators (for details see Table S7).

### Factors associated with the estimated discrepancies

#### Self-rated CHE without support from the objective indicators

Table 3 shows that larger household size, living with the elderly or young children, lower educational attainment, lower household income, lower perceived social class, unemployment, rural residency, higher per capita governmental budget on health, and western region were associated with higher odds of self-rated CHE without support from the objective indicators. Compared with those without social health insurance, the respondents covered by the Urban Employee Basic Medical Insurance (UEBMI) or the New Rural Cooperative Medical Scheme (NRCMS) were more likely to have self-rated CHE without support from objective indicators. The modelling results were largely consistent across the four objective estimation approaches. The crude odds ratios are presented in supplementary Table S8, see online supplementary material.

**Table 3.** Adjusted odds ratios (AORs) of factors associated with self-rated CHE without support from objective indicators

	Model 1 (*n* = 27 601) Budget share method vs self-rating		Model 2 (*n* = 27 749) Actual food spending method vs self-rating		Model 3 (*n* = 26 434) Partial normative food spending method vs self-rating		Model 4 (*n* = 26 282) Standard subsistence spending method vs self-rating	
	AOR^a^	[95 % CI]	AOR	[95 % CI]	AOR	[95 % CI]	AOR	[95 % CI]
**Demographic factor**								
Household size								
Small (ref.)								
Middle	1.072*	[0.992 to 1.158]	1.059	[0.981 to 1.142]	1.066	[0.985 to 1.154]	1.052	[0.972 to 1.138]
Large	1.330***	[1.217 to 1.453]	1.335***	[1.224 to 1.456]	1.347***	[1.230 to 1.475]	1.295***	[1.182 to 1.419]
Gender ratio								
Lower (ref.)								
Middle	0.951	[0.886 to 1.020]	0.943	[0.880 to 1.011]	0.958	[0.891 to 1.030]	0.958	[0.891 to 1.030]
Higher	1.013	[0.945 to 1.085]	1.014	[0.947 to 1.086]	1.040	[0.968 to 1.116]	1.022	[0.953 to 1.097]
**Needs factor**								
Aged care need								
No (ref.)								
Yes	1.158***	[1.067 to 1.257]	1.160***	[1.070 to 1.258]	1.160***	[1.066 to 1.262]	1.169***	[1.074 to 1.273]
Childcare need								
No (ref.)								
Yes	1.069*	[0.989 to 1.154]	1.051	[0.974 to 1.134]	1.048	[0.968 to 1.135]	1.054	[0.973 to 1.141]
**Socioeconomic factor**								
Highest educational attainment								
Up to primary school	1.225***	[1.063 to 1.413]	1.200**	[1.042 to 1.383]	1.193**	[1.028 to 1.385]	1.146*	[0.983 to 1.336]
Middle school	1.185***	[1.093 to 1.284]	1.190***	[1.100 to 1.288]	1.196***	[1.102 to 1.298]	1.196***	[1.103 to 1.298]
Vocational training	1.124***	[1.034 to 1.221]	1.106**	[1.019 to 1.200]	1.128***	[1.037 to 1.227]	1.141***	[1.050 to 1.240]
Tertiary degree (Ref.)								
Per capita household income								
Lowest	2.062***	[1.848 to 2.300]	2.046***	[1.836 to 2.279]	1.800***	[1.609 to 2.013]	1.855***	[1.654 to 2.081]
Lower	1.829***	[1.649 to 2.028]	1.828***	[1.651 to 2.025]	1.648***	[1.483 to 1.831]	1.785***	[1.609 to 1.980]
Middle	1.664***	[1.506 to 1.839]	1.639***	[1.485 to 1.810]	1.598***	[1.446 to 1.766]	1.660***	[1.504 to 1.833]
Higher	1.428***	[1.295 to 1.574]	1.444***	[1.312 to 1.590]	1.394***	[1.266 to 1.536]	1.453***	[1.321 to 1.599]
Highest (Ref.)								
Perceived social class								
Upper (ref.)								
Middle	1.545**	[1.009 to 2.367]	1.537**	[1.009 to 2.339]	1.618**	[1.042 to 2.514]	1.566**	[1.014 to 2.417]
Lower	2.678***	[1.744 to 4.111]	2.715***	[1.779 to 4.142]	2.801***	[1.798 to 4.361]	2.736***	[1.768 to 4.235]
Proportion of non-working age members								
Lower (ref.)								
Middle	0.981	[0.907 to 1.062]	0.990	[0.915 to 1.070]	0.990	[0.913 to 1.074]	0.992	[0.915 to 1.075]
Higher	1.049	[0.947 to 1.162]	1.039	[0.939 to 1.150]	1.076	[0.969 to 1.196]	1.057	[0.951 to 1.174]
Unemployment of working-age members								
No (ref.)								
Yes	1.238***	[1.167 to 1.314]	1.247***	[1.177 to 1.322]	1.265***	[1.191 to 1.344]	1.279***	[1.204 to 1.358]
Residency								
Urban (ref.)								
Rural	1.103***	[1.035 to 1.175]	1.100***	[1.034 to 1.171]	1.1***	[1.030 to 1.174]	1.090***	[1.022 to 1.163]
Alignment of residency with household registration								
Aligned (Ref.)								
Non-aligned	0.941	[0.859 to 1.031]	0.918*	[0.838 to 1.005]	0.929	[0.847 to 1.019]	0.943	[0.862 to 1.033]
Health insurance^b^								
None (ref.)								
UEBMI	1.142**	[1.026 to 1.271]	1.158***	[1.041 to 1.287]	1.182***	[1.061 to 1.316]	1.152***	[1.035 to 1.281]
URBMI	1.037	[0.920 to 1.168]	1.022	[0.908 to 1.151]	1.052	[0.932 to 1.188]	1.041	[0.924 to 1.174]
GMI	1.066	[0.806 to 1.410]	1.090	[0.825 to 1.441]	1.094	[0.825 to 1.450]	1.069	[0.809 to 1.412]
NRCMS	1.169***	[1.077 to 1.268]	1.191***	[1.099 to 1.291]	1.169***	[1.075 to 1.272]	1.171***	[1.077 to 1.273]
Others	0.968	[0.833 to 1.125]	0.955	[0.823 to 1.107]	0.944	[0.809 to 1.102]	0.922	[0.790 to 1.075]
Regional factor								
Per capita provincial governmental budget on health								
Lower (ref.)								
Middle	1.017	[0.950 to 1.088]	1.014	[0.949 to 1.084]	1.010	[0.942 to 1.082]	1.018	[0.950 to 1.090]
Higher	1.115**	[1.014 to 1.226]	1.113**	[1.014 to 1.222]	1.115**	[1.012 to 1.228]	1.090*	[0.989 to 1.201]
Regional location of province								
Eastern (Ref.)								
Central	0.959	[0.896 to 1.027]	0.978	[0.914 to 1.046]	0.989	[0.921 to 1.061]	0.939*	[0.875 to 1.007]
Western	1.071*	[0.994 to 1.154]	1.082**	[1.005 to 1.165]	1.115***	[1.033 to 1.204]	1.073*	[0.994 to 1.158]
Year								
2013 (ref.)								
2017	0.900***	[0.831 to 0.974]	0.931*	[0.860 to 1.007]	0.913**	[0.842 to 0.991]	0.910**	[0.839 to 0.987]
2019	0.994	[0.916 to 1.078]	1.007	[0.929 to 1.092]	1.024	[0.942 to 1.113]	1.012	[0.930 to 1.100]
2021	0.844***	[0.774 to 0.920]	0.869***	[0.798 to 0.946]	0.849***	[0.776 to 0.928]	0.841***	[0.770 to 0.920]
Pseudo R-square	0.043		0.044		0.039		0.040	
AIC	29 536.009		30 202.701		28 082.711		28 142.537	

* *P* < 0.1,

** *P* < 0.05,

*** *P* < 0.01;

a AOR = adjusted odds ratio, CI = confidence interval;

b UEBMI = Urban Employee Basic Medical Insurance, URBMI = Urban Resident Basic Medical Insurance, GMI = Government Medical Insurance, NRCMS = New Rural Cooperative Medical Scheme.

#### CHE classified by the objective indicators without backup from self-rating

Table 4 shows that higher levels of per capita household income, higher perceived social class, and reporting no unemployed working-age members were associated with higher odds of being classified with CHE by the objective indicators without backup from self-rating. Those who resided in the more affluent eastern region were more likely to have CHE classified by the objective indicators without backup from self-rating. The crude odds ratios are presented in Supplementary Table S9, see online supplementary material.

**Table 4.** Adjusted odds ratios (AORs) of factors associated with households classified with CHE by the objective indicators without backup from self-rating

	Model 1 (*n* = 4 088) Budget share method vs self-rating		Model 2 (*n* = 3 417) Actual food spending method vs self-rating		Model 3 (*n* = 5 195) Partial normative food spending method vs self-rating		Model 4 (*n* = 5 422) Standard subsistence spending method vs self-rating	
	AOR^a^	[95 % CI]	AOR	[95 % CI]	AOR	[95 % CI]	AOR	[95 % CI]
Demographic factor								
Household size								
Small (ref.)								
Middle	1.157	[0.952 to 1.406]	1.007	[0.810 to 1.252]	1.088	[0.922 to 1.283]	1.143*	[0.975 to 1.341]
Large	0.980	[0.796 to 1.207]	0.978	[0.777 to 1.230]	1.008	[0.845 to 1.203]	0.972	[0.820 to 1.153]
Gender ratio								
Lower (Ref.)								
Middle	1.067	[0.902 to 1.262]	1.033	[0.861 to 1.240]	1.089	[0.946 to 1.253]	1.038	[0.907 to 1.189]
Higher	0.957	[0.804 to 1.140]	0.904	[0.744 to 1.098]	1.012	[0.874 to 1.172]	0.981	[0.852 to 1.129]
Needs factor								
Aged care need								
No (ref.)								
Yes	1.122	[0.922 to 1.365]	1.187	[0.952 to 1.479]	1.028	[0.871 to 1.214]	1.064	[0.910 to 1.244]
Childcare need								
No (ref.)								
Yes	1.160	[0.956 to 1.408]	1.180	[0.949 to 1.466]	1.086	[0.922 to 1.279]	1.021	[0.874 to 1.192]
Socioeconomic factor								
Highest educational attainment								
Up to primary school	0.999	[0.741 to 1.348]	1.060	[0.765 to 1.470]	0.886	[0.685 to 1.147]	0.986	[0.775 to 1.253]
Middle school	0.922	[0.746 to 1.140]	1.013	[0.798 to 1.285]	0.966	[0.806 to 1.157]	1.018	[0.852 to 1.217]
Vocational training	1.029	[0.819 to 1.293]	0.967	[0.745 to 1.255]	0.989	[0.812 to 1.204]	1.030	[0.843 to 1.258]
Tertiary degree (Ref.)								
Per capita household income								
Lowest	0.593***	[0.441 to 0.797]	0.468***	[0.341 to 0.643]	0.559***	[0.422 to 0.742]	1.072	[0.793 to 1.450]
Lower	0.675***	[0.503 to 0.907]	0.541***	[0.393 to 0.745]	0.719**	[0.544 to 0.951]	1.049	[0.773 to 1.423]
Middle	0.711**	[0.528 to 0.958]	0.558***	[0.404 to 0.769]	0.771*	[0.580 to 1.024]	0.958	[0.699 to 1.313]
Higher	0.705**	[0.524 to 0.949]	0.553***	[0.399 to 0.766]	0.657***	[0.490 to 0.881]	0.809	[0.583 to 1.123]
Highest (Ref.)								
Perceived social class								
Upper (ref.)								
Middle	0.709	[0.278 to 1.808]	0.569	[0.225 to 1.436]	0.926	[0.408 to 2.100]	0.717	[0.327 to 1.573]
Lower	0.387**	[0.151 to 0.991]	0.324**	[0.128 to 0.823]	0.527	[0.232 to 1.201]	0.391**	[0.178 to 0.860]
Proportion of non-working age members								
Lower (Ref.)								
Middle	1.099	[0.896 to 1.347]	1.032	[0.822 to 1.294]	1.020	[0.860 to 1.210]	1.010	[0.859 to 1.189]
Higher	1.042	[0.807 to 1.346]	0.964	[0.725 to 1.280]	1.031	[0.832 to 1.278]	0.916	[0.747 to 1.123]
Unemployment of working-age members								
No (ref.)								
Yes	0.807***	[0.699 to 0.932]	0.819**	[0.698 to 0.962]	0.752***	[0.666 to 0.849]	0.728***	[0.648 to 0.819]
Residency								
Urban (ref.)								
Rural	1.110	[0.947 to 1.302]	1.067	[0.897 to 1.270]	0.975	[0.851 to 1.116]	1.019	[0.786 to 1.322]
Alignment of residency with household registration								
Aligned (Ref.)								
Non-aligned	1.006	[0.756 to 1.340]	0.860	[0.623 to 1.186]	0.878	[0.685 to 1.125]	-^§^	-
Health insurance^b^								
None (ref.)								
UEBMI	1.249	[0.926 to 1.683]	1.493**	[1.083 to 2.058]	1.157	[0.883 to 1.517]	1.130	[0.851 to 1.501]
URBMI	0.971	[0.702 to 1.344]	0.915	[0.640 to 1.309]	0.994	[0.760 to 1.299]	0.897	[0.677 to 1.188]
GMI	0.558	[0.221 to 1.411]	0.919	[0.406 to 2.084]	0.834	[0.413 to 1.684]	0.610	[0.284 to 1.311]
NRCMS	1.103	[0.905 to 1.343]	1.131	[0.912 to 1.402]	0.943	[0.803 to 1.109]	1.008	[0.861 to 1.180]
Others	1.825***	[1.309 to 2.544]	1.685***	[1.163 to 2.442]	1.324*	[0.989 to 1.773]	1.231	[0.920 to 1.647]
Regional factor								
Per capita provincial governmental budget on health								
Lower (Ref.)								
Middle	0.851*	[0.720 to 1.006]	0.911	[0.760 to 1.093]	0.889	[0.772 to 1.024]	0.906	[0.790 to 1.039]
Higher	1.010	[0.797 to 1.280]	1.025	[0.790 to 1.329]	0.965	[0.789 to 1.181]	0.933	[0.768 to 1.133]
Regional location of province								
Eastern (ref.)								
Central	0.944	[0.801 to 1.111]	0.929	[0.776 to 1.113]	1.096	[0.954 to 1.258]	0.888*	[0.776 to 1.017]
Western	0.756***	[0.625 to 0.913]	0.760***	[0.619 to 0.934]	0.971	[0.827 to 1.140]	0.877*	[0.753 to 1.023]
Year								
2013 (ref.)								
2017	1.069	[0.875 to 1.307]	1.063	[0.852 to 1.325]	0.913	[0.772 to 1.081]	0.865*	[0.740 to 1.011]
2019	1.275**	[1.035 to 1.570]	1.215*	[0.964 to 1.530]	1.160*	[0.975 to 1.381]	1.046	[0.888 to 1.232]
2021	1.242*	[0.995 to 1.551]	1.306**	[1.024 to 1.665]	1.229**	[1.025 to 1.472]	0.994	[0.834 to 1.186]
Pseudo R-square	0.035		0.036		0.031		0.025	
AIC	4 903.282		4 087.031		6 712.182		7 211.764	

* Notes: *P*  0.1,

** *P* < 0.05,

*** *P* < 0.01;

a AOR = adjusted odds ratio, CI = confidence interval;

b UEBMI = Urban Employee Basic Medical Insurance, URBMI = Urban Resident Basic Medical Insurance, GMI = Government Medical Insurance, NRCMS = New Rural Cooperative Medical Scheme.

§ cannot generate results.

#### Robustness check

In the regression models, we transformed several independent variables, including household size, gender ratio, per capita household income, proportion of non-working age members, and per capita governmental budget on health, into categorical variables. However, to the best of our knowledge, there is insufficient theoretical justification for assuming either a linear or non-linear association between these dependent variables and the discrepancies between self-rated and objective CHE. Consequently, we re-ran the regression analyses using continuous forms of these variables (results can be found in supplementary Tables S10 and S11, see online supplementary material). The main findings remained consistent, demonstrating the robustness of our results.

## Discussion

Our study utilised five indicators to estimate CHE in China using CSS data from 2013 to 2021, comparing the results between objective and subjective indicators. The trend in CHE incidence remained consistent over this period, regardless of the indicator employed. However, the prevalence of self-rated CHE was significantly higher than that estimated by objective indicators, suggesting that the latter may underestimate CHE in certain instances. Notably, the normative subsistence spending method produced the lowest prevalence of situations where self-rated CHE lacked support from objective indicators, indicating that essential needs may extend beyond food to include housing, utilities, and other factors in self-rated assessments of CHE.

The most concerning finding of this study is the association between lower socioeconomic status and higher odds of self-rated CHE lacking support from objective indicators. Conversely, households with higher socioeconomic status were more likely to be classified as experiencing CHE by objective indicators without corresponding self-reports. This discrepancy may stem from the inherent limitations of objective CHE indicators. Wealthier households, despite a significant proportion of OOP healthcare expenditure relative to their total budgets, often have greater capacity to mobilise other resources, such as savings, to maintain a reasonable standard of living. Additionally, these households typically possess a stronger ability to recover from financial shocks, a factor not accounted for in any objective CHE indicators. Similar to previous studies, our findings indicate a potential ‘pro-rich’ bias in objective CHE indicators (Van Doorslaer et al. 2007, Grépin et al. 2020, Trakinsky et al. 2020). Furthermore, we found that unemployment and living with elderly or young children were associated with higher odds of self-rated CHE lacking support from objective indicators.

We propose several ways to improve CHE monitoring and future research. First, the self-rating indicator should be considered a complementary tool to current CHE indicators in assessing financial protection progress, allowing for a more comprehensive understanding of households’ financial hardship. Although changing routine data reporting is challenging and time-consuming to implement, individual perceptions of financial difficulties can be included in national health surveys and other household surveys (e.g. China Family Panel Studies). Second, broadening the scope of essential needs may enhance the validity of objective methods, better reflecting social realities and norms. Presently, objective indicators typically define essential needs as food, housing, or utilities. However, the significant discrepancies observed between objective indicators and self-rated CHE potentially indicate that the existing definition of essential needs—especially when primarily focused on food—may be insufficient, potentially overlooking other needs that individuals consider essential. This observation is further supported by findings indicating that the standard subsistence spending method, which includes housing and utilities as essential, produced the smallest discrepancy in self-rated CHE without corroboration from objective indicators (Cylus et al. 2018). The delineation of essential needs in CHE calculations goes beyond technical considerations, reflecting prevailing social norms. The acceptability of a household’s OOP healthcare expenditures related to specific necessities likely varies significantly across different countries and cultures. Therefore, we advocate for further research aimed at defining the appropriate scope of essential needs in diverse international contexts and investigating the practical efficacy of self-assessment methodologies in the design, oversight, and evaluation of health financial protection systems. Lastly, the observed association between lower socioeconomic status and higher odds of self-rated CHE, without corresponding support from objective indicators, suggests that existing objective measures may underestimate CHE for households with lower socioeconomic status due to their constrained essential needs (Trakinsky et al. 2020). This finding underscores the necessity of adjusting the thresholds for poorer households when utilising existing objective indicators to monitor CHE.

This study has several limitations that should be acknowledged. First, households experiencing the financial burden of OOP healthcare expenditures may reduce their healthcare utilisation, which could partly explain the observed lower incidence of CHE generated by objective methods compared to self-reported measures. However, this observation does not fully account for the instances where objective indicators lack support from self-rated responses. Second, we cannot rule out the possibility that respondents may under-report their expenditures or over-report their hardships in the survey to qualify for additional social aid (Banerjee et al. 2020). Nevertheless, the significant associations observed between socioeconomic status and the discrepancies between self-rated and objective indicators suggest that reporting bias alone cannot entirely explain these discrepancies. It is worth noting that CSS respondents were first asked to recall their household OOP healthcare expenditures, followed by questions about self-rated CHE. The presence of ∼40 questions between these two inquiries minimises the potential for over-reporting driven by a desire to validate their self-rated CHE.

Third, the data utilised in this study does not come from a panel survey; therefore, we were unable to control for the fixed effects of individual factors, which are necessary to account for potential bias arising from unobserved individual-specific characteristics. However, each wave of the CSS draws on a nationally representative sample for that year. Although we did not employ a causal inference design in this study, this was partially due to the nature of the CSS not being a panel survey. Importantly, this study does not aim to ascertain the causal effects of socioeconomic status on the discrepancies between self-rated and objective CHE. Fourth, due to data unavailability, we did not include health conditions and healthcare utilisation in the regression analyses. Fifth, the self-rating scale comprises only one item, which causes more reporting bias than a multi-item scale. We advocate for research aimed at developing valid multi-item scales to measure subjective CHE. Insights can be drawn from the 12-item Comprehensive Score for Financial Toxicity, which has been widely used to assess both the objective and subjective financial toxicity associated with cancer treatment (Azzani et al. 2024). Sample items include statements such as, ‘I worry about the financial problems I will have in the future as a result of my illness or treatment’ (de Souza et al. 2017). Future studies can adapt this scale to align with the definition of CHE. Furthermore, while the standard subsistence spending method resulted in the lowest prevalence of cases where self-rated CHE lacked support from objective indicators, its reliance on detailed expenditure data for each budget item limits its practical applicability. Finally, due to data constraints, this study was based solely on data from one country, which may restrict the external validity of the findings. Nonetheless, this limitation warrants attention, as China is one of the most populous developing countries, and similar challenges are likely to be encountered in other LMICs. Currently, there is a lack of research on discrepancies between objective and subjective assessments of CHE, thus we were unable to compare our results with previous studies.

## Conclusion

To the best of our knowledge, this study is the first to identify discrepancies between objective and subjective approaches in estimating CHE. The observed discrepancies are relatively high. While we cannot argue that self-rating is superior to objective methods, the socioeconomic factors contributing to these discrepancies should be a significant concern in discussions surrounding financial protection indicators. Vulnerable populations, such as the elderly, young children, and individuals with low socioeconomic status, are those that financing systems should prioritise for protection. However, a financial protection policy based solely on objective CHE indicators risks failing to adequately safeguard these groups. Moreover, a small percentage of households classified as experiencing CHE by objective indicators may not actually endure financial hardship, further complicating the assessment of financial protection and the effectiveness of healthcare financing systems.

## Supplementary Material

czae115_Supp

## Data Availability

The Chinese Social Survey data are available at http://css.cssn.cn/css_sy/xmjs/. It is open for application for researchers. The China national data online database is publicly available at https://data.stats.gov.cn/english/.

## Reflexivity statement

All authors of this article are scholars in universities. One is female and two are male. One is a PhD student, one an early career researcher, and one a senior researcher.

## Patient and public involvement statement

The data used in the study were not collected by the authors. The authors have no data on respondents contact information. Thus, it was not appropriate or possible to involve patients or the public in the design, conduct, reporting, or dissemination plans of our research.
