# Supplementary material for: The discrepancy between objective and subjective assessments of catastrophic health expenditure: evidence from China
Source: Health Policy Plan. 2024 Dec 2;40(3):331–45. doi: 10.1093/heapol/czae115 (PMC11886810; doi:10.1093/heapol/czae115)
Supplement: czae115_Supp [file czae115_supp.zip › Supplementary materials-RR.pdf]

Supplementary Materials

Table of Contents

Figure S1. Sample selection process 2

Table S1. Full questions to measure self-rated CHE and objective CHE indicators 3

Table S2. Dependent variables 5

Table S3. Cut-off points of independent variables 6

Table S4. ICCs of self-rated CHE without support from objective indicators and CHE being classified by the objective indicators without backup from self-rating 7

Table S5. AICs of logistic models and multilevel models 7

Table S6. Incidence of household catastrophic health expenditure from 2013 to 2021 8

Table S7. Consistency in estimations of household catastrophic health expenditure between self-rating and the objective indicators in China, 2013-2021 9

Table S8. Factors associated with self-rated CHE without support from objective indicators (crude odds ratio, COR) 10

Table S9. Factors associated with households classified with CHE by the objective indicators without backup from self-rating (crude odds ratio, COR) 13

Table S10. Factors associated with self-rated CHE without support from objective indicators (using continuous independent variables) 16

Table S11. Factors associated with households classified with CHE by the objective indicators without backup from self-rating (using continuous independent variables) 19

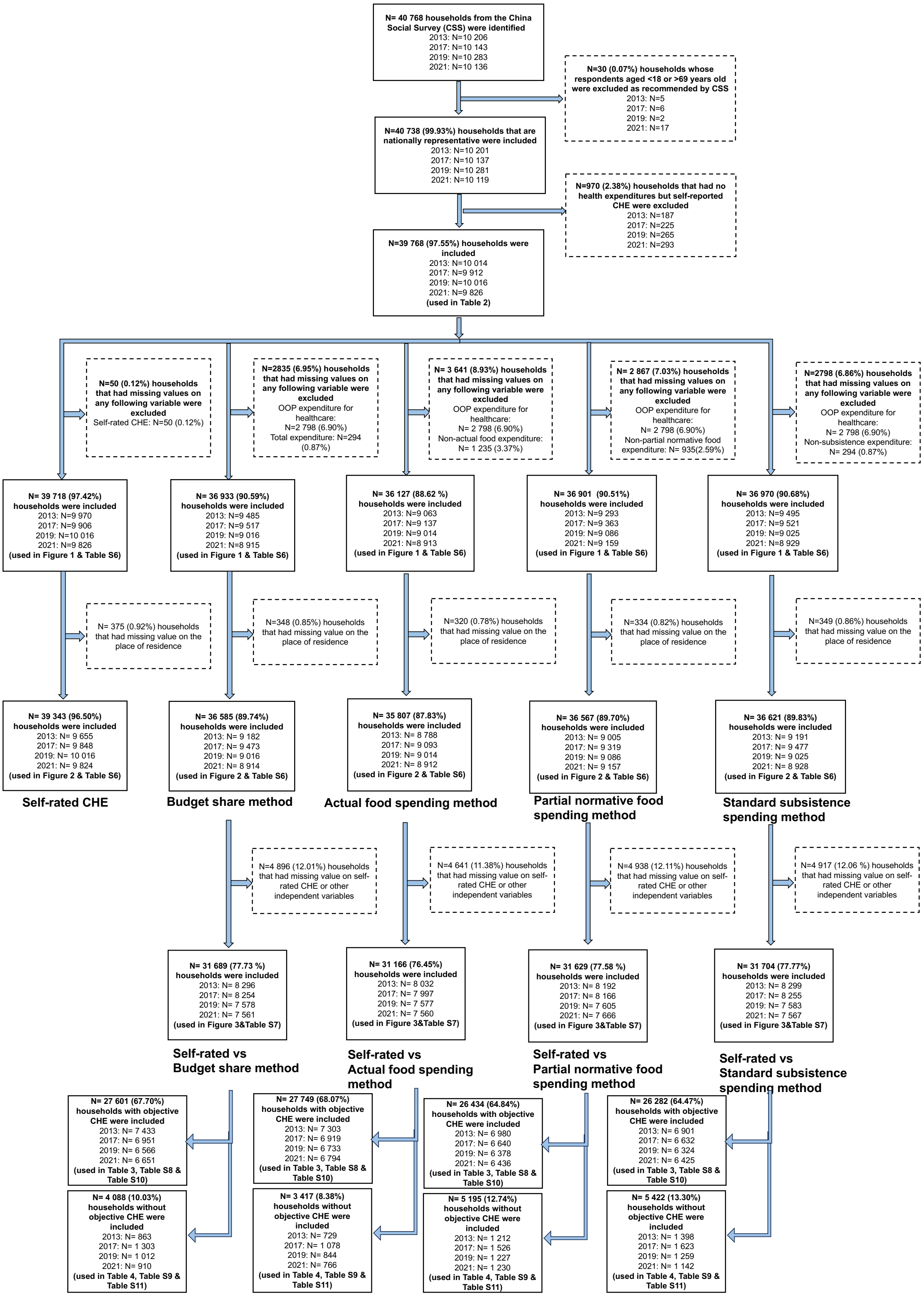

Figure S1. Sample selection process

Notes: In this figure, the denominator of all proportions is the initially sample size (40 768).

Table S1. Full questions to measure self-rated CHE and objective CHE indicators

Year	Objective CHE indicators<br>Full question	Sequence	Self-rating CHE<br>Full question	Sequence
2013	Please tell me your household expenditures on following items in the last year<br>• Total<br>• **Food**<br>• Clothes<br>• **Housing rent**<br>• Down payment and mortgage loan for housing<br>• **Utility (gas, water, electricity)**<br>• Furniture appliances, automobile, and housekeeping<br>• **Healthcare (including reimbursement)**<br>• Telecommunication<br>• Travel and transport<br>• Education<br>• Cultural and recreation activities<br>• Care for family members who do not live together<br>• Gifts to relatives or friends<br>• Others<br><br>Please tell me your household income on the following items in the last year? (one item is '**h. health insurance reimbursement**')<br><br>**Out-of-pocket payment healthcare expenditure**= Healthcare expenditure (including reimbursement)- health insurance reimbursement	Third section of the questionnaire, called 'household economic situation'	Did you or your family have the following problem in the last year? (one option is '**the out-of-pocket payment healthcare expenditure is too high to be affordable**')	Fourth section of the questionnaire called 'media usage and self-evaluation on life'
2017	No changes	No changes	No changes	No changes
2019	No changes	No changes	No changes	No changes
2021	No changes	No changes	No changes	No changes

Notes: The sequence and full questions of the survey items that we used in this study between 2013 and 2021 have remained the same.

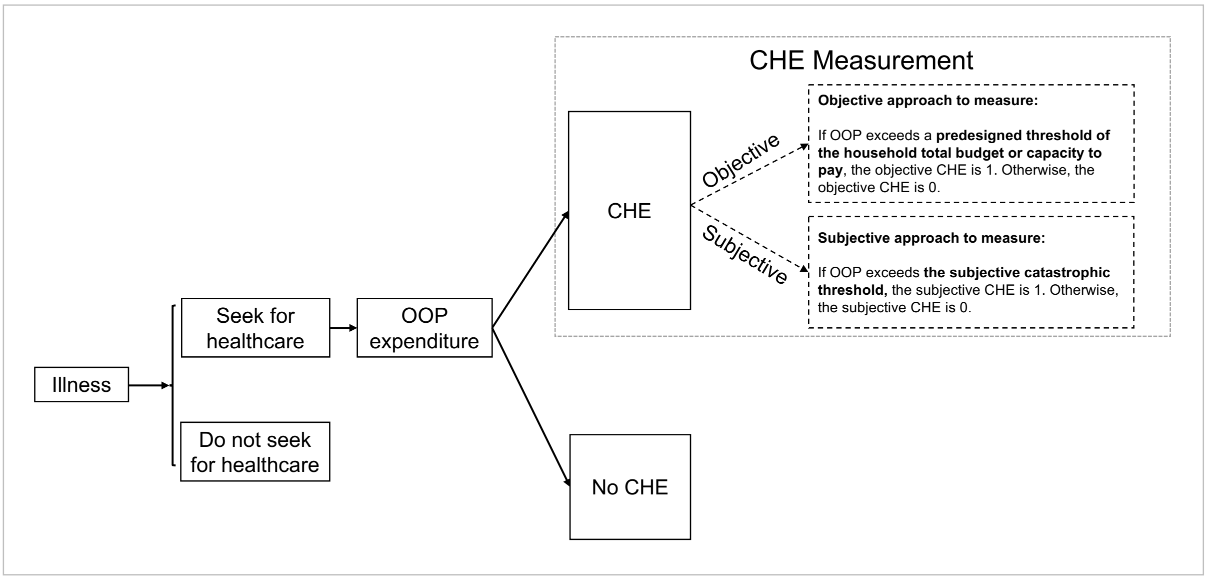

Figure S2. Two approaches to measure catastrophic health expenditure (CHE)

Table S2. Dependent variables

Type of discrepancy	Dependent variable	Dependent variable (1=Yes)	Dependent variable (0=No)	Regression model
Self-rated CHE without support from objective indicators	Self-rated CHE without support from objective indicators (budget share method vs self-rating)	Self-rated CHE= Yes<br>Objective CHE (budget share method) = No	Self-rated CHE= Yes<br>Objective CHE (budget share method) = Yes	Table 3 Model 1
	Self-rated CHE without support from objective indicators (actual food spending method vs self-rating)	Self-rated CHE= Yes<br>Objective CHE (actual food spending method) = No	Self-rated CHE= Yes<br>Objective CHE (actual food spending method) = Yes	Table 3 Model 2
	Self-rated CHE without support from objective indicators (partial normative food spending method vs self-rating)	Self-rated CHE= Yes<br>Objective CHE (partial normative food spending method) = No	Self-rated CHE= Yes<br>Objective CHE (partial normative food spending method) = Yes	Table 3 Model 3
	Self-rated CHE without support from objective indicators (normative subsistence spending method vs self-rating)	Self-rated CHE= Yes<br>Objective CHE (normative subsistence spending method) = No	Self-rated CHE= Yes<br>Objective CHE (normative subsistence spending method) = Yes	Table 3 Model 4
Households classified with CHE by the objective indicators without backup from self-rating	Households classified with CHE by the objective indicators (budget share method vs self-rating) without backup from self-rating	Self-rated CHE= No<br>Objective CHE (budget share method) = Yes	Self-rated CHE= No<br>Objective CHE (budget share method) = No	Table 4 Model 1
	Households classified with CHE by the objective indicators (actual food spending method vs self-rating) without backup from self-rating	Self-rated CHE= No<br>Objective CHE (actual food spending method) = Yes	Self-rated CHE= No<br>Objective CHE (actual food spending method) = No	Table 4 Model 2
	Households classified with CHE by the objective indicators (partial normative food spending method vs self-rating) without backup from self-rating	Self-rated CHE= No<br>Objective CHE (partial normative food spending method) = Yes	Self-rated CHE= No<br>Objective CHE (partial normative food spending method) = No	Table 4 Model 3
	Households classified with CHE by the objective indicators (normative subsistence spending method vs self-rating) without backup from self-rating	Self-rated CHE= No<br>Objective CHE (normative subsistence spending method) = Yes	Self-rated CHE= No<br>Objective CHE (normative subsistence spending method) = No	Table 4 Model 4

Table S3. Cut-off points of independent variables

Variable	Category	2013 [min, max]	2017 [min, max]	2019 [min, max]	2021 [min, max]
Household size	Small	[1,3]	[1,3]	[1,4]	[1,4]
	Medium	[4,5]	[4,5]	[5,5]	[5,5]
	Large	[6,16]	[6,25]	[6,30]	[6,25]
Gender ratio	Lower	[0,40]	[0,40]	[0,40]	[0,40]
	Middle	(40, 50]	(40, 50]	(40, 50]	(40, 50]
	Higher	(50, 100]	(50, 100]	(50, 100]	(50, 100]
Per capita household income	Lowest	[0, 4 125]	[0, 3 833.333]	[0, 3 500]	[0, 4 800]
	Low	[4 133.333, 7 920]	[3 840, 8 000]	[3 516.667, 8 610]	[4 828.571, 10 000]
	Middle	[7 924, 12 500]	[8 016.667, 13 666.67]	[8 614.286, 15 200]	[10 010.67, 17 500]
	High	[12 512, 20 000]	[13 680, 24 000]	[15 214.29, 27 500]	[17 520, 32 666.67]
	Highest	[20 037.14, 2 666 667]	[24 013.33, 1 050 000]	[27 520, 3 333 333]	[32 857.14, 1 345 000]
Proportion of non-working age members	Lower	[0,0]	[0,0]	[0, 16.66667]	[0, 16.66667]
	Middle	[10, 33.33333]	[8.333333, 33.33333]	[17.85714, 33.33333]	[18.18182, 33.33333]
	Higher	[36.36364, 100]	[35.71429, 100]	[35.29412, 100]	[35.29412, 100]
Per capita governmental budget on health	Lower	[498.5225, 580.8838]	[780.6818, 1 003.088]	[852.3264, 1 129.529]	[944.0766, 1 247.181]
	Middle	[585.6568, 675.9727]	[1 034.293, 1 146.868]	[1 130.674, 1 300.957]	[1 255.296, 1 464.128]
	Higher	[680.3223, 1 299.435]	[1 147.016, 2 687.679]	[1 334.079, 3 408.587]	[1 511.375, 3 149.18]

Table S4. ICCs of self-rated CHE without support from objective indicators and CHE being classified by the objective indicators without backup from self-rating

Variable	Budget share vs self-rating	Actual food spending vs self-rating	Partial normative food spending vs self-rating	Standard subsistence spending vs self-rating
Self-rated CHE without support from objective indicators	0.012	0.011	0.011	0.011
CHE being classified by the objective indicators without backup from self-rating	0.010	0.007	0.007	0.013

Table S5. AICs of logistic models and multilevel models

Model	Budget share vs self-rating	Actual food spending vs self-rating	Partial normative food spending vs self-rating	Standard subsistence spending vs self-rating
**Self-rated CHE without support from objective indicators**				
Logistic model	29 536.009	30 202.701	28 082.711	28 142.537
Multilevel model	29 506.504	30 182.333	28 056.237	28 118.652
**Households classified with CHE by objective indicators without backup from self-rating**				
Logistic model	4 903.282	4 087.031	6 712.182	7 211.764
Multilevel model	4 903.282	4 089.008	6 712.182	7 207.995

Table S6. Incidence of household catastrophic health expenditure from 2013 to 2021

Year	Self-rating		Budget share		Actual food spending		Partial normative food spending		Normative subsistence spending	
	N	%	N	%	N	%	N	%	N	%
2013										
Urban	1 081	24.91	299	7.30	262	6.64	348	8.61	347	8.47
Rural	1 852	34.84	735	14.45	608	12.56	1 054	21.24	1 316	25.84
Total	3 047	30.56	1 071	11.29	899	9.92	1 446	15.56	1 724	18.16
2017										
Urban	1 471	35.99	751	19.18	641	17.16	893	23.26	968	24.71
Rural	1 848	32.08	882	15.87	727	13.57	996	18.18	1 118	20.11
Total	3 340	33.72	1 641	17.24	1 376	15.06	1 895	20.24	2 092	21.97
2019										
Urban	1 546	27.34	612	12.21	523	10.44	697	13.78	635	12.65
Rural	1 632	37.42	821	20.50	703	17.56	1 020	25.32	1 165	29.09
Total	3 178	31.73	1 433	15.89	1 226	13.60	1 717	18.90	1 800	19.94
2021										
Urban	929	21.44	348	8.90	316	8.08	529	13.06	349	8.91
Rural	1 855	33.79	922	18.43	804	16.07	1 248	24.45	1 263	25.20
Total	2 786	28.35	1 270	14.25	1 120	12.57	1 778	19.41	1 612	18.05
2013-2021										
Urban	5 027	27.30	2 010	11.87	1 742	10.49	2 467	14.52	2 299	13.56
Rural	7 187	34.34	3 360	17.10	2 842	14.80	4 318	22.06	4 862	24.72
Total	12 351	31.10	5 415	14.66	4 621	12.79	6 836	18.53	7 228	19.55

Notes: The sample size varied due to the removal of responses containing missing values.

Table S7. Consistency in estimations of household catastrophic health expenditure between self-rating and the objective indicators in China, 2013-2021

Year	Budget share vs self-rating		Actual food spending vs self-rating		Partial normative food spending vs self-rating		Normative subsistence spending vs self-rating	
	N	%†	N	%	N	%	N	%
**Panel 1: Self-rated CHE without support from objective indicators (self-rating CHE=yes & objective CHE=no)**								
2013	2 178	23.07	2 156	23.90	1 960	21.18	1 969	20.83
2017	2 054	21.59	2 129	23.31	1 908	20.38	1 912	20.09
2019	1 977	21.93	2 105	23.35	1 918	21.11	1 888	20.92
2021	1 714	19.23	1 822	20.44	1 609	17.57	1 626	18.21
Total	7 923	21.48	8 212	22.76	7 395	20.06	7 395	20.03
**Panel 2: Households classified with CHE by objective indicators without backup from self-rating (self-rating CHE=no & objective CHE=yes)**								
2013	310	3.28	246	2.73	520	5.62	750	7.93
2017	458	4.81	379	4.15	610	6.52	767	8.06
2019	482	5.35	403	4.47	694	7.64	760	8.42
2021	417	4.68	375	4.21	765	8.35	671	7.51
Total	1 667	4.52	1 403	3.89	2 589	7.02	2 948	7.98
**Panel 3: Households without CHE identified by both objective and self-rating indicators (self-rating CHE=no & objective CHE=no)**								
2013	6 196	65.62	5 969	66.16	5 849	63.22	5 766	61.00
2017	5 820	61.17	5 630	61.63	5 558	59.37	5 515	57.94
2019	5 606	62.18	5 683	63.05	5 451	59.99	5 337	59.14
2021	5 931	66.53	5 971	66.99	5 772	63.02	5 691	63.74
Total	23 553	63.85	23 253	64.44	22 630	61.40	22 309	60.42
**Panel 4: Household with CHE identified by both objective and self-rating indicators (self-rating CHE=yes & objective CHE=yes)**								
2013	758	8.03	651	7.22	923	9.98	967	10.23
2017	1 182	12.42	997	10.91	1 285	13.73	1 324	13.91
2019	951	10.55	823	9.13	1 023	11.26	1 040	11.52
2021	853	9.57	745	8.36	1 013	11.06	941	10.54
Total	3 744	10.15	3 216	8.91	4 244	11.51	4 272	11.57

Notes: † In this table, for all panels, the proportion column (%) represents the comparison between the sample size in the previous column (N) and the total sample size of the corresponding year. Each year's total sample size used in this table is presented in Supplementary Figure S1.

Table S8. Factors associated with self-rated CHE without support from objective indicators (crude odds ratio, COR)

	Model 1 Budget share vs self-rating		Model 2 Actual food spending method vs self-rating		Model 3 Partial normative food spending method vs self-rating		Model 4 Standard subsistence spending method vs self-rating	
	COR†	[95 % CI]	COR	[95 % CI]	COR	[95 % CI]	COR	[95 % CI]
**Predisposing factor**								
Household size								
Small (Ref.)								
Middle	1.301***	[1.223 to 1.383]	1.303***	[1.226 to 1.384]	1.283***	[1.205 to 1.367]	1.283***	[1.205 to 1.367]
Large	1.846***	[1.735 to 1.965]	1.842***	[1.732 to 1.959]	1.815***	[1.702 to 1.936]	1.769***	[1.658 to 1.887]
Gender ratio								
Lower (Ref.)								
Middle	1.083**	[1.018 to 1.153]	1.073**	[1.009 to 1.141]	1.091***	[1.023 to 1.164]	1.081**	[1.013 to 1.152]
Higher	1.092***	[1.026 to 1.162]	1.095***	[1.030 to 1.164]	1.116***	[1.047 to 1.190]	1.099***	[1.031 to 1.172]
**Needs factor**								
Aged care need								
No (Ref.)								
Yes	1.492***	[1.414 to 1.575]	1.487***	[1.409 to 1.568]	1.516***	[1.434 to 1.603]	1.491***	[1.409 to 1.577]
Childcare need								
No (Ref.)								
Yes	1.259***	[1.190 to 1.332]	1.238***	[1.171 to 1.309]	1.240***	[1.169 to 1.314]	1.239***	[1.169 to 1.313]
**Enabling factor**								
Highest educational attainment								
Up to primary school	1.821***	[1.622 to 2.044]	1.790***	[1.594 to 2.009]	1.712***	[1.516 to 1.934]	1.631***	[1.437 to 1.852]
Middle school	1.580***	[1.478 to 1.690]	1.587***	[1.486 to 1.696]	1.535***	[1.433 to 1.643]	1.539***	[1.438 to 1.648]
Vocational training	1.299***	[1.205 to 1.400]	1.283***	[1.192 to 1.382]	1.283***	[1.189 to 1.384	1.307***	[1.212 to 1.410]
Tertiary degree (Ref.)								

Per capita household income								
Lowest	3.039***	[2.782 to 3.320]	3.089***	[2.830 to 3.372]	2.655***	[2.426 to 2.906]	2.549***	[2.319 to 2.803]
Lower	2.503***	[2.295 to 2.729]	2.539***	[2.331 to 2.767]	2.234***	[2.046 to 2.440]	2.373***	[2.177 to 2.588]
Middle	2.142***	[1.963 to 2.337]	2.127***	[1.950 to 2.319]	2.027***	[1.858 to 2.211]	2.097***	[1.925 to 2.285]
Higher	1.622***	[1.483 to 1.773]	1.657***	[1.517 to 1.810]	1.585***	[1.451 to 1.730]	1.639***	[1.503 to 1.788]
Highest (Ref.)								
Perceived social class								
Upper (Ref.)								
Middle	1.571**	[1.064 to 2.321]	1.548**	[1.053 to 2.276]	1.625**	[1.087 to 2.430]	1.660**	[1.102 to 2.499]
Lower	3.037***	[2.053 to 4.494]	3.054***	[2.074 to 4.498]	3.059***	[2.042 to 4.582]	3.139***	[2.080 to 4.736]
Proportion of non-working age members								
Lower (Ref.)								
Middle	1.296***	[1.218 to 1.379]	1.299***	[1.222 to 1.380]	1.290***	[1.210 to 1.375]	1.284***	[1.205 to 1.369]
Higher	1.596***	[1.496 to 1.702]	1.561***	[1.464 to 1.665]	1.604***	[1.501 to 1.715]	1.575***	[1.473 to 1.684]
Unemployment of working-age members								
No (Ref.)								
Yes	1.309***	[1.241 to 1.381]	1.325***	[1.257 to 1.397]	1.338	[1.266 to 1.413]	1.362***	[1.290 to 1.439]
Residency								
Urban (Ref.)								
Rural	1.355***	[1.287 to 1.427]	1.363***	[1.295 to 1.434]	1.311***	[1.243 to 1.382]	1.270***	[1.204 to 1.339]
Alignment of residency with household registration								
Aligned (Ref.)								
Non-aligned	0.765***	[0.705 to 0.829]	0.752***	[0.693 to 0.815]	0.778***	[0.717 to 0.845]	0.806***	[0.744 to 0.874]
Health insurance‡								
None (Ref.)								
UEBMI	0.808***	[0.739 to 0.884]	0.808***	[0.739 to 0.884]	0.881***	[0.804 to 0.964]	0.873***	[0.798 to 0.955]

URBMI	0.895**	[0.805 to 0.994]	0.883**	[0.795 to 0.980]	0.930	[0.835 to 1.036]	0.945	[0.850 to 1.051]
GMI	0.710***	[0.555 to 0.909]	0.740**	[0.580 to 0.945]	0.773**	[0.604 to 0.989]	0.760**	[0.597 to 0.969]
NRCMS	1.353***	[1.262 to 1.451]	1.373***	[1.281 to 1.471]	1.357***	[1.262 to 1.459]	1.332***	[1.239 to 1.433]
Others	0.886*	[0.776 to 1.011]	0.892*	[0.783 to 1.016]	0.888*	[0.775 to 1.018]	0.872**	[0.761 to 0.999]
Per capita provincial governmental budget on health								
lower (Ref.)								
Middle	1.070**	[1.012 to 1.132]	1.068**	[1.011 to 1.129]	1.070**	[1.010 to 1.133]	1.071**	[1.011 to 1.135]
Higher	1.102**	[1.019 to 1.191]	1.106**	[1.024 to 1.195]	1.129***	[1.042 to 1.224]	1.097**	[1.012 to 1.189]
Regional location of province								
Eastern (Ref.)								
Central	1.065**	[1.002 to 1.132]	1.110***	[1.045 to 1.179]	1.083**	[1.017 to 1.154]	1.034	[0.971 to 1.101]
Western	1.286***	[1.208 to 1.368]	1.320***	[1.241 to 1.403]	1.316***	[1.234 to 1.403]	1.266***	[1.187 to 1.350]
Year								
2013 (Ref.)								
2017	1.004	[0.936 to 1.077]	1.047	[0.976 to 1.123]	1.024	[0.952 to 1.102]	1.015	[0.944 to 1.092]
2019	1.003	[0.935 to 1.077]	1.025	[0.956 to 1.100]	1.050	[0.976 to 1.130]	1.036	[0.963 to 1.115]
2021	0.822***	[0.765 to 0.884]	0.845***	[0.786 to 0.908]	0.832***	[0.772 to 0.897]	0.837***	[0.776 to 0.902]

Notes: †COR=Crude odds ratio; CI=Confidence Interval; * p<0.1, **p<0.05, *** p<0.01; ‡UEBMI=Urban Employee Basic Medical Insurance; URBMI=Urban Resident Basic Medical Insurance; GMI=Government Medical Insurance; NRCMS=New Rural Cooperative Medical Scheme

Table S9. Factors associated with households classified with CHE by the objective indicators without backup from self-rating (crude odds ratio, COR)

	Model 1 Budget share vs self-rating		Model 2 Actual food spending method vs self-rating		Model 3 Partial normative food spending method vs self-rating		Model 4 Standard subsistence spending method vs self-rating	
	COR†	[95 % CI]	COR	[95 % CI]	COR	[95 % CI]	COR	[95 % CI]
**Predisposing factor**								
Household size								
Small (Ref.)								
Middle	1.023	[0.887 to 1.180]	0.915	[0.782 to 1.071]	0.953	[0.844 to 1.076]	1.124*	[0.999 to 1.264]
Large	1.001	[0.874 to 1.147]	0.930	[0.803 to 1.078]	0.926	[0.825 to 1.039]	1.029	[0.921 to 1.149]
Gender ratio								
Lower (Ref.)								
Middle	1.079	[0.941 to 1.238]	1.062	[0.916 to 1.230]	1.050	[0.935 to 1.179]	0.967	[0.865 to 1.080]
Higher	0.968	[0.835 to 1.123]	0.943	[0.801 to 1.109]	1.010	[0.892 to 1.144]	0.933	[0.828 to 1.051]
**Needs factor**								
Aged care need								
No (Ref.)								
Yes	1.053	[0.938 to 1.183]	1.093	[0.964 to 1.239]	0.922	[0.835 to 1.018]	0.965	[0.878 to 1.061]
Childcare need								
No (Ref.)								
Yes	1.114	[0.977 to 1.270]	1.001	[0.865 to 1.158]	1.027	[0.919 to 1.147]	1.031	[0.927 to 1.146]
**Enabling factor**								
Highest educational attainment								
Up to primary school	0.823*	[0.658 to 1.029]	0.808*	[0.635 to 1.029]	0.626***	[0.516 to 0.760]	0.855*	[0.712 to 1.027]
Middle school	0.857*	[0.724 to 1.016]	0.866	[0.718 to 1.044]	0.757***	[0.657 to 0.872]	0.956	[0.827 to 1.105]
Vocational training	1.027	[0.847 to 1.245]	0.966	[0.779 to 1.197]	0.864*	[0.734 to 1.017]	1.004	[0.849 to 1.189]
Tertiary degree (Ref.)								

Per capita household income								
Lowest	0.501***	[0.401 to 0.626]	0.423***	[0.335 to 0.535]	0.471***	[0.377 to 0.588]	0.853	[0.669 to 1.086]
Lower	0.582***	[0.462 to 0.733]	0.479***	[0.374 to 0.612]	0.620***	[0.494 to 0.777]	0.862	[0.670 to 1.108]
Middle	0.664***	[0.521 to 0.846]	0.535***	[0.413 to 0.694]	0.694***	[0.547 to 0.880]	0.888	[0.681 to 1.157]
Higher	0.654***	[0.508 to 0.840]	0.554***	[0.423 to 0.726]	0.618***	[0.480 to 0.797]	0.769*	[0.580 to 1.020]
Highest (Ref.)								
Perceived social class								
Upper (Ref.)								
Middle	0.613	[0.279 to 1.348]	0.475*	[0.219 to 1.031]	0.780	[0.380 to 1.600]	0.659	[0.343 to 1.266]
Lower	0.303***	[0.137 to 0.669]	0.235***	[0.108 to 0.513]	0.384***	[0.187 to 0.791]	0.353***	[0.183 to 0.680]
Proportion of non-working age members								
Lower (Ref.)								
Middle	1.084	[0.934 to 1.257]	0.954	[0.810 to 1.122]	0.944	[0.835 to 1.067]	1.012	[0.898 to 1.141]
Higher	1.120	[0.969 to 1.295]	1.043	[0.894 to 1.217]	0.920	[0.815 to 1.039]	0.956	[0.850 to 1.075]
Unemployment of working-age members								
No (Ref.)								
Yes	0.845**	[0.742 to 0.961]	0.866**	[0.750 to 1.000]	0.784***	[0.704 to 0.873]	0.734***	[0.661 to 0.814]
Residency								
Urban (Ref.)								
Rural	1.027	[0.911 to 1.158]	0.979	[0.860 to 1.115]	0.928	[0.838 to 1.028]	1.087	[0.982 to 1.203]
Alignment of residency with household registration								
Aligned (Ref.)								
Non-aligned	0.953	[0.747 to 1.216]	0.936	[0.718 to 1.219]	1.006	[0.822 to 1.231]	0.975	[0.785 to 1.212]
Health insurance‡								
None (Ref.)								
UEBMI	1.402***	[1.119 to 1.756]	1.593***	[1.258 to 2.018]	1.256**	[1.024 to 1.541]	1.100	[0.884 to 1.367]

URBMI	0.989	[0.760 to 1.288]	1.053	[0.796 to 1.393]	1.093	[0.882 to 1.355]	0.951	[0.757 to 1.194]
GMI	0.997	[0.539 to 1.844]	1.545	[0.870 to 2.743]	1.257	[0.758 to 2.085]	0.779	[0.429 to 1.412]
NRCMS	1.046	[0.895 to 1.223]	1.036	[0.875 to 1.225]	0.904	[0.796 to 1.026]	1.046	[0.924 to 1.184]
Others	1.721***	[1.313 to 2.257]	1.646***	[1.222 to 2.219]	1.416***	[1.121 to 1.788]	1.274**	[1.006 to 1.614]
Per capita provincial governmental budget on health								
lower (Ref.)								
Middle	0.859**	[0.755 to 0.978]	0.923	[0.803 to 1.062]	0.892**	[0.799 to 0.994]	0.902*	[0.813 to 1.001]
Higher	0.837*	[0.697 to 1.004]	0.933	[0.767 to 1.135]	0.911	[0.781 to 1.063]	0.842**	[0.726 to 0.976]
Regional location of province								
Eastern (Ref.)								
Central	0.887*	[0.775 to 1.016]	0.895	[0.772 to 1.037]	1.038	[0.925 to 1.165]	0.862***	[0.770 to 0.965]
Western	0.687***	[0.594 to 0.795]	0.718***	[0.614 to 0.841]	0.856**	[0.758 to 0.968]	0.832***	[0.741 to 0.933]
Year								
2013 (Ref.)								
2017	0.947	[0.799 to 1.124]	1.006	[0.833 to 1.215]	0.843**	[0.729 to 0.973]	0.747***	[0.656 to 0.851]
2019	1.239**	[1.044 to 1.471]	1.296***	[1.073 to 1.565]	1.204**	[1.042 to 1.391]	0.942	[0.824 to 1.077]
2021	1.195**	[1.002 to 1.426]	1.332***	[1.099 to 1.614]	1.340***	[1.162 to 1.546]	0.919	[0.801 to 1.055]

Notes: †COR=crude odds ratio; CI=Confidence Interval; * p<0.1, **p<0.05, *** p<0.01; ‡UEBMI=Urban Employee Basic Medical Insurance; URBMI=Urban Resident Basic Medical Insurance; GMI=Government Medical Insurance; NRCMS=New Rural Cooperative Medical Scheme.

Table S10. Factors associated with self-rated CHE without support from objective indicators (using continuous independent variables)

	Model 1 (N= 27 601) Budget share method vs self-rating		Model 2 (N=27 749) Actual food spending method vs self-rating		Model 3 (N=26 434) Partial normative food spending method vs self-rating		Model 4 (N=26 282) Standard subsistence spending method vs self-rating	
	AOR†	[95 % CI]	AOR	[95 % CI]	AOR	[95 % CI]	AOR	[95 % CI]
**Predisposing factor**								
Household size	1.083***	[1.064 to 1.102]	1.076***	[1.058 to 1.095]	1.076***	[1.057 to 1.096]	1.076***	[1.056 to 1.096]
Proportion of female members in a household	1.002*	[1.000 to 1.003]	1.001	[1.000 to 1.003]	1.002*	[1.000 to 1.004]	1.002*	[1.000 to 1.004]
**Needs factor**								
Aged care need								
No (Ref.)								
Yes	1.209***	[1.115 to 1.311]	1.205***	[1.112 to 1.306]	1.208***	[1.111 to 1.312]	1.210***	[1.113 to 1.316]
Childcare need								
No (Ref.)								
Yes	1.071*	[0.992 to 1.155]	1.057	[0.980 to 1.140]	1.055	[0.975 to 1.141]	1.054	[0.975 to 1.141]
**Enabling factor**								
Highest educational attainment								
Up to primary school	1.394***	[1.209 to 1.608]	1.349***	[1.170 to 1.555]	1.324***	[1.141 to 1.538]	1.285***	[1.102 to 1.499]
Middle school	1.252***	[1.155 to 1.357]	1.263***	[1.166 to 1.368]	1.257***	[1.158 to 1.365]	1.264***	[1.165 to 1.372]
Vocational training	1.137***	[1.047 to 1.235]	1.132***	[1.043 to 1.230]	1.147***	[1.055 to 1.248]	1.164***	[1.071 to 1.265]
Tertiary degree (Ref.)								
Per capita household income	0.993***	[0.991 to 0.995]	0.991***	[0.989 to 0.993]	0.995***	[0.993 to 0.996]	0.994***	[0.992 to 0.996]
Perceived social class								
Upper (Ref.)								
Middle	1.504*	[0.984 to 2.300]	1.593**	[1.034 to 2.452]	1.590**	[1.023 to 2.471]	1.542*	[0.998 to 2.382]
Lower	2.745***	[1.791 to 4.206]	2.875***	[1.863 to 4.436]	2.843***	[1.825 to 4.430]	2.796***	[1.805 to 4.329]

Proportion of non-working age members	0.999	[0.997 to 1.001]	0.999	[0.997 to 1.001]	1.000	[0.998 to 1.002]	1.000	[0.998 to 1.002]
Unemployment of working-age members								
Yes	1.229***	[1.159 to 1.303]	1.252***	[1.181 to 1.327]	1.264***	[1.190 to 1.343]	1.278***	[1.203 to 1.357]
Residency								
Urban (Ref.)								
Rural	1.106***	[1.039 to 1.178]	1.108***	[1.041 to 1.179]	1.104***	[1.035 to 1.177]	1.095***	[1.027 to 1.167]
Alignment of residency with household registration								
Aligned (Ref.)								
Non-aligned	0.914*	[0.835 to 1.001]	0.890**	[0.812 to 0.975]	0.905**	[0.826 to 0.993]	0.919*	[0.839 to 1.006]
Health insurance‡								
None (Ref.)								
UEBMI	1.087	[0.978 to 1.208]	1.106*	[0.995 to 1.230]	1.128**	[1.014 to 1.255]	1.097*	[0.987 to 1.219]
URBMI	1.008	[0.895 to 1.135]	0.996	[0.884 to 1.121]	1.025	[0.908 to 1.157]	1.016	[0.902 to 1.145]
GMI	1.016	[0.770 to 1.342]	1.036	[0.782 to 1.373]	1.043	[0.787 to 1.381]	1.020	[0.773 to 1.346]
NRCMS	1.186***	[1.094 to 1.286]	1.209***	[1.116 to 1.310]	1.185***	[1.090 to 1.289]	1.185***	[1.090 to 1.287]
Others	0.960	[0.827 to 1.115]	0.941	[0.810 to 1.093]	0.934	[0.800 to 1.090]	0.907	[0.778 to 1.058]
Per capita provincial governmental budget on health	1.000	[1.000 to 1.000]	1.000	[1.000 to 1.000]	1.000	[1.000 to 1.000]	1.000	[1.000 to 1.000]
Regional location of province								
Eastern (Ref.)								
Central	0.964	[0.901 to 1.032]	0.980	[0.916 to 1.048]	0.995	[0.928 to 1.068]	0.947	[0.883 to 1.015]
Western	1.112***	[1.035 to 1.194]	1.119***	[1.043 to 1.201]	1.147***	[1.066 to 1.234]	1.102***	[1.025 to 1.186]
Year								
2013 (Ref.)								

2017	0.899**	[0.821 to 0.984]	0.934	[0.854 to 1.021]	0.902**	[0.822 to 0.990]	0.903**	[0.823 to 0.991]
2019	1.008	[0.911 to 1.115]	1.028	[0.930 to 1.136]	1.021	[0.920 to 1.132]	1.021	[0.921 to 1.132]
2021	0.882**	[0.785 to 0.990]	0.906*	[0.808 to 1.016]	0.860**	[0.764 to 0.968]	0.866**	[0.769 to 0.975]
Pseudo R-square	0.040		0.041		0.037		0.037	
AIC	29 620.617		30 280.507		28 144.802		28 206.002	

Notes: †AOR =Adjusted odds ratio; CI=Confidence Interval; * p<0.1, **p<0.05, *** p<0.01; ‡UEBMI=Urban Employee Basic Medical Insurance; URBMI=Urban Resident Basic Medical Insurance; GMI=Government Medical Insurance; NRCMS=New Rural Cooperative Medical Scheme.

Table S11. Factors associated with households classified with CHE by the objective indicators without backup from self-rating (using continuous independent variables)

	Model 1 (N=4 088) Budget share method vs self-rating		Model 2 (N=3 417) Actual food spending method vs self-rating		Model 3 (N=5 195) Partial normative food spending method vs self-rating		Model 4 (N=5 422) Standard subsistence spending method vs self-rating	
	AOR†	[95 % CI]	AOR	[95 % CI]	AOR	[95 % CI]	AOR	[95 % CI]
**Predisposing factor**								
Household size	0.997	[0.957 to 1.039]	1.000	[0.956 to 1.045]	0.983	[0.950 to 1.018]	1.011	[0.980 to 1.043]
Proportion of female members in a household	1.000	[0.996 to 1.004]	0.997	[0.992 to 1.001]	0.998	[0.995 to 1.002]	0.999	[0.996 to 1.003]
**Needs factor**								
Aged care need								
No (Ref.)								
Yes	1.107	[0.910 to 1.348]	1.174	[0.942 to 1.463]	0.994	[0.842 to 1.173]	1.033	[0.883 to 1.210]
Childcare need								
No (Ref.)								
Yes	1.148	[0.950 to 1.389]	1.149	[0.928 to 1.422]	1.079	[0.919 to 1.267]	0.982	[0.843 to 1.143]
**Enabling factor**								
Highest educational attainment								
Up to primary school	0.941	[0.698 to 1.270]	1.055	[0.760 to 1.465]	0.825	[0.637 to 1.070]	0.986	[0.775 to 1.256]
Middle school	0.913	[0.739 to 1.129]	1.021	[0.804 to 1.297]	0.946	[0.789 to 1.134]	1.027	[0.858 to 1.228]
Vocational training	1.019	[0.811 to 1.280]	0.968	[0.746 to 1.257]	0.986	[0.810 to 1.201]	1.031	[0.844 to 1.259]
Tertiary degree (Ref.)								
Per capita household income	1.006**	[1.001 to 1.010]	1.009***	[1.003 to 1.014]	1.005**	[1.001 to 1.009]	0.998	[0.993 to 1.002]
Perceived social class								
Upper (Ref.)								
Middle	0.753	[0.297 to 1.906]	0.599	[0.239 to 1.503]	0.921	[0.408 to 2.082]	0.732	[0.335 to 1.600]

Lower	0.403*	[0.158 to 1.027]	0.335**	[0.133 to 0.845]	0.511	[0.226 to 1.159]	0.405**	[0.185 to 0.888]
Proportion of non-working age members	1.001	[0.996 to 1.006]	0.999	[0.994 to 1.004]	1.003	[0.998 to 1.007]	0.999	[0.995 to 1.003]
Unemployment of working-age members								
Yes	0.808***	[0.699 to 0.932]	0.817**	[0.696 to 0.958]	0.757***	[0.671 to 0.855]	0.730***	[0.649 to 0.820]
Residency								
Urban (Ref.)								
Rural	1.095	[0.935 to 1.284]	1.049	[0.882 to 1.248]	0.976	[0.853 to 1.117]	1.005	[0.880 to 1.147]
Alignment of residency with household registration								
Aligned (Ref.)								
Non-aligned	1.022	[0.769 to 1.359]	0.885	[0.643 to 1.218]	0.890	[0.695 to 1.139]	1.005	[0.776 to 1.303]
Health insurance‡								
None (Ref.)								
UEBMI	1.276	[0.950 to 1.713]	1.578***	[1.151 to 2.165]	1.180	[0.903 to 1.541]	1.086	[0.821 to 1.436]
URBMI	0.991	[0.717 to 1.370]	0.941	[0.660 to 1.343]	1.013	[0.776 to 1.323]	0.882	[0.667 to 1.168]
GMI	0.584	[0.232 to 1.472]	0.995	[0.442 to 2.236]	0.849	[0.421 to 1.712]	0.599	[0.279 to 1.288]
NRCMS	1.108	[0.910 to 1.349]	1.124	[0.907 to 1.393]	0.944	[0.804 to 1.109]	1.010	[0.863 to 1.182]
Others	1.819***	[1.305 to 2.534]	1.693***	[1.170 to 2.450]	1.336*	[0.999 to 1.787]	1.209	[0.904 to 1.617]
Per capita provincial governmental budget on health	1.000	[1.000 to 1.000]	1.000	[1.000 to 1.001]	1.000**	[1.000 to 1.000]	1.000**	[1.000 to 1.000]
Regional location of province								
Eastern (Ref.)								
Central	0.967	[0.822 to 1.138]	0.956	[0.799 to 1.143]	1.120	[0.976 to 1.285]	0.911	[0.796 to 1.043]
Western	0.698***	[0.583 to 0.836]	0.710***	[0.583 to 0.864]	0.884	[0.761 to 1.028]	0.807***	[0.700 to 0.932]
Year								

2013 (Ref.)								
2017	0.988	[0.787 to 1.241]	0.962	[0.748 to 1.236]	0.835*	[0.689 to 1.012]	0.792**	[0.662 to 0.949]
2019	1.087	[0.848 to 1.395]	1.037	[0.786 to 1.368]	0.993	[0.805 to 1.225]	0.891	[0.728 to 1.089]
2021	1.017	[0.762 to 1.356]	1.064	[0.775 to 1.460]	1.009	[0.795 to 1.281]	0.828	[0.658 to 1.042]
Pseudo R-square		0.032		0.033		0.029		0.024
AIC		4 901.934		4 082.762		6 716.649		7 208.165

Notes: †AOR =adjusted odds ratio; CI=Confidence Interval; * p<0.1, **p<0.05, *** p<0.01; ‡UEBMI=Urban Employee Basic Medical Insurance; URBMI=Urban Resident Basic Medical Insurance; GMI=Government Medical Insurance; NRCMS=New Rural Cooperative Medical Scheme.
